# Genetic and Environmental Influences on Free Sugar and Nonnutritive Sweetener Intake in Toddlerhood and Middle Childhood Using Data from the Gemini Twin Cohort: Findings from the SWEET Project

**DOI:** 10.1016/j.tjnut.2026.101586

**Published:** 2026-05-08

**Authors:** Lisa Heggie, Zeynep Nas, Rana Conway, Alison Fildes, Andrea Smith, Edith Feskens, Jason CG Halford, Joanne A Harrold, Anne Raben, Clare H Llewellyn

**Affiliations:** 1 Department of Behavioural Science and Health, University College London, London, United Kingdom; 2 School of Psychology, Faculty of Medicine and Health, University of Leeds, Leeds, United Kingdom; 3 School of Clinical Medicine, Institute of Metabolic Science, MRC Epidemiology Unit, University of Cambridge, Cambridge, United Kingdom; 4 Division of Human Nutrition and Health, Wageningen University & Research, Wageningen, The Netherlands; 5 Department of Psychology, Institute of Population Health, University of Liverpool, Liverpool, United Kingdom; 6 Department of Nutrition, Exercise and Sports, SCIENCE, University of Copenhagen, Copenhagen, Denmark; 7 Department for Clinical and Translational Research, Copenhagen University Hospital–Steno Diabetes Center Copenhagen, Herlev, Denmark

**Keywords:** diet quality, childhood nutrition, nonsugar sweeteners, nonnutritive sweeteners, free sugar

## Abstract

**Background:**

Exceeding recommended free sugar (FS) intake is linked to poorer metabolic health. Nonnutritive sweeteners (NNS) offer an alternative to FS, and emerging evidence suggests that many children regularly consume them. Understanding the etiology of variation in FS and NNS intake in childhood can inform population-level strategies to reduce sugar consumption.

**Objectives:**

This study aimed to examine genetic and environmental influences on variation in FS and NNS intakes in toddlerhood and middle childhood, and investigate stability and change over time.

**Methods:**

FS (as % energy intake) and NNS (categorized as non-, low-, and high consumers) were estimated using 3-d dietary data at 21 mo (*n* = 2592) with repeated measures in a subsample at 7 y (*n* = 592) from British twins in the Gemini cohort. Longitudinal Cholesky twin models estimated genetic and environmental influences on FS and NNS intakes at both ages.

**Results:**

Shared environmental factors strongly influenced both FS and NNS intakes at 21 mo [FS: 0.88; 95% confidence interval (CI): 0.86, 0.90; NNS: 0.99; 95% CI: 0.99, 1] and 7 y (FS: 0.58; 95% CI: 0.45, 0.69; NNS: 0.92; 95% CI: 0.86, 1), but the effect decreased significantly over time for FS. There was no significant genetic influence on NNS at either age; genetic influence on FS intake increased significantly from toddlerhood (FS: 0.09; 95% CI: 0.08, 0.11) to middle childhood (FS: 0.33; 95% CI: 0.22, 0.47). Small longitudinal correlations indicated greater change than stability in intakes over time for FS (*r* = 0.28, *P* < 0.001) and NNS *(r =* 0.35, *P* < 0.001), explained predominantly by novel shared environmental influences in middle childhood and the emergence of novel genetic influence on FS.

**Conclusions:**

Shared environmental factors predominantly shape individual differences in FS and NNS intake during early life. Although increasing genetic influence from toddlerhood to middle childhood reflects growing autonomy in dietary choices for FS intake, variation in NNS intake remains overwhelmingly environmentally driven across both developmental periods. Findings emphasize the need for both age-specific public health interventions and food industry legislation to shape FS and NNS intakes in childhood.

## Introduction

Childhood dietary habits are critical determinants of health, and the consumption of free sugars (FS) and nonnutritive sweeteners (NNS) is of particular concern [[1], [2], [3], [4]]. Intake of FS above recommended guidelines is linked with poorer metabolic health [[5], [6], [7]]. The WHO recommends limiting FS consumption to <10% of daily energy intake and to <5% for additional health benefits; however, many children exceed these recommendations [8]. Globally, children (<18 y) consume between 11% and 28% of their daily energy intake from FS, raising concerns about longer-term obesity risk and poorer metabolic health [[9], [10], [11], [12], [13], [14], [15]].

NNS provide few or no calories and are increasingly used as sugar substitutes in processed foods and beverages, e.g., aspartame, which is ∼200 times sweeter than sucrose [16]. This substitution could potentially reduce childhood FS intake, and studies indicate that 17% to 50% of children aged 3 to 6 y may consume NNS from beverages and dairy [17,18]. A recent WHO meta-analysis informed recommendation cautioning against the use of NNS to support weight management or reduce noncommunicable disease risk in adults and children. However, because this was based largely on observational studies and not randomized controlled clinical studies, more research is required [19,20]. Understanding the determinants of childhood FS intake may aid population-level interventions to reduce sugar intake. Investigation into the gene–environment interplay underlying NNS intake may provide further clarity to research on NNS and health.

Twin studies establish the relative contribution of genetic and environmental factors to variation in behaviors. They partition individual differences in behavior into 3 sources of variance: genetic influences (“heritability”), shared environmental influences, and nonshared environmental influences. Although twin studies have established that childhood eating behaviors, including food preferences, appetitive traits, and food neophobia, are under moderate-to-high genetic control [21,22], the relative contribution of genes and the environment to variation in specific aspects of dietary intake in infancy and toddlerhood remains understudied [23,24].

Data from the British Gemini twin cohort (*n* = 1216 twin pairs) highlighted the importance of shared environmental factors for variation in intakes of macronutrients, food groups, and beverages at 21 mo of age, with virtually no role of genetic factors [23]. The proportion of variance in energy from macronutrients explained by shared environmental influences was 86% for fats and carbohydrates and 87% for protein, with very low genetic influence (8%–10%). For “added sugars and confectionery,” the shared environmental influence was estimated at 91%; genetic influence was only 5%. In contrast, data from 7-y-old United States twins in the MacArthur Longitudinal Twin Study (*n* = 396 twin pairs) showed much lower shared environment contributions to individual differences in confectionery intake (37% for boys and 58% for girls), and a sizable genetic influence (41% for boys and 27% for girls) [25]. To date, no information is available about the genetic and environmental determinants of variation in FS and NNS intakes in toddlerhood or childhood, nor on their contribution to stability and change in intake across this developmental transition period.

This study therefore aimed to establish: *1*) the proportion of variation in FS and NNS intake explained by genetic and environmental factors in toddlerhood and middle childhood; *2*) whether there are significant differences in genetic and environmental influence on variation in FS and NNS in toddlerhood and middle childhood; and *3*) the genetic and environmental contributions to stability and change in intakes of FS and NNS from toddlerhood to middle childhood.

## Methods

This study is part of the “Sweeteners and sweetness enhancers: Impact on health, obesity, safety and sustainability” (SWEET) project (http://www.sweetproject.eu), a large, European Commission–funded program of international research aiming to establish the longer-term benefits and risks of substituting NNS for sugar in the context of obesity, safety, sustainability, and health.

### Participants

Participants were from Gemini, a population-based British twin cohort established to investigate genetic and environmental influences on early childhood weight trajectories with a focus on appetite and family environment [26]. Families with twins born in England and Wales between March and December 2007 were identified and contacted by the Office for National Statistics (*n* = 6754) and asked to provide permission to be contacted by the Gemini research team. About half of the sample consented to be contacted (*n* = 3435; 51%), of whom 2402 families completed baseline questionnaires (70% response rate). Ethical approval was granted for the study by the University College London (UCL) Committee for the Ethics of Non-National Health Service Human Research. Written informed consent was provided by all families.

To date, there have been 11 waves of data collection in Gemini. The current study used data from the baseline questionnaire (wave 1, when the cohort was ∼8 mo old), the zygosity questionnaire (wave 5, 30 mo of age), and 2 rounds of dietary data collection (waves 3 and 9, 21 mo and 7 y of age, respectively).

### FS intake and NNS intake

Dietary data were collected via parent-reported, 3-d diet diaries in toddlerhood (*n* = 2592; mean age: 20.9 mo; SD: 1.1) with repeat measures in a subsample in middle childhood (*n* = 592; mean age: 7.1 y; SD: 0.2). Methods for dietary data collection and coding of the 21-mo diet diaries have been described in detail in a previously published study investigating dietary intake by toddlers of Gemini [27]. The Gemini diaries collected at 7 y of age was coded by UCL using the same dietary coding software, coding rules, and data quality control processes. Toddlerhood dietary intakes in Gemini are generally comparable with those reported by the United Kingdom’s National Diet and Nutrition Survey (NDNS) for the same age group [27]. In brief, when the twins were ∼21 mo old, all participating Gemini families were invited to complete diet diaries for both twins over 3 d (*n* = 2393 families). Fifty-seven percent of the sample returned diet diaries at this time point (*n* = 1357 families). When the twins were ∼7 y of age, a subsample of families who returned diet diaries at 21 mo were invited to complete a second diet diary (*n* = 1845 families; 77% of baseline sample). Seventeen percent of the invited sample returned diet diaries (*n* = 309 families). Twin pairs with a minimum of 2 d of dietary data at 21 mo and/or at 7 y of age was included in the current sample (Supplementary Figure 1). Dietary data were coded using the dietary assessment software Diet In Nutrients Out, which draws nutritional data from the United Kingdom Food Standards Agency’s NDNS nutritional databank [28,29].

Dietary FS intake was estimated using a method previously published by Kibblewhite et al. [30], adapted to meet the United Kingdom’s definition of FS by inclusion of all sugar in pureed fruits [31,32]. In brief, FS refers to all added sugars and naturally occurring sugars present in honey, syrup, and pure fruit juice, whereas added sugar refers only to sugars added to consumable items during product processing. Both FS and added sugar fall into the category of total sugar, which includes all sugars present in an item, regardless of source. A novel method was developed to quantify NNS intake from beverages, in milligrams, from self- or parent-reported population-level dietary data and applied to the Gemini sample, described in detail elsewhere [33]. Daily energy, FS, and NNS intakes were calculated at both 21 mo and 7 y of age as a mean of dietary data collected over ≥2 of 3 d. FS intake was described as a percentage of total energy intake (%EI). Intakes of individual NNS types were estimated and aggregated as “total NNS,” referred to as NNS intake from herein, in milligrams per day (mg/d). NNS intake was highly skewed, with a large proportion of the sample consuming none. It was therefore characterized as an ordinal variable, including nonconsumers (0), low consumers (1), and high consumers (2). We used a median split to classify consumers into “low consumers” (21 mo: <27.89 mg; 7 y: <36.92 mg) and “high consumers” (21 mo: >27.89 mg; 7 y: >36.92 mg).

### Zygosity, age, and sex

Parents provided information on the sex, date of birth, gestational age, and birthweight of their twins in the baseline questionnaire (mean age of twins at baseline questionnaire: 8.2 mo; SD: 2.2). The age of children at each data collection was calculated by subtracting the date of birth from the date of the final day of diet diary completion. Parents also reported their ethnic background in the baseline questionnaire. Maternal ethnicity was categorized as either “White” or “non-White.” At baseline, parents provided information on several socioeconomic indicators, including the highest educational qualifications of the parents, current occupations of both parents, total annual household income, postcode, home ownership status, number of bedrooms in the home, and number of cars. The socioeconomic data were aggregated to generate a composite score of socioeconomic status (SES), described elsewhere [34].

Zygosity was assessed using a validated parent-reported questionnaire measure [35,36] completed at baseline and 29 mo, with additional validation using DNA.

### Analyses

Sociodemographic characteristics of the dietary data samples were compared with the baseline sample using Pearson’s χ^2^ tests for categorical variables and independent samples *t*-tests for continuous variables.

The classical twin design relies on the known genetic difference between identical twins (monozygotic, MZ) who share all their genetic material, and nonidentical (dizygotic, DZ) twins who share, on average, 50% of their segregating genetic material. The 2 sets of twin pairs are assumed to have similar environmental exposure; the shared environment is therefore correlated equally across MZ and DZ twins. If, therefore, MZ twin pairs are more alike than DZ twin pairs for a measured phenotype (e.g., FS intake), genetics is likely to explain some of the variance in that phenotype. If, however, the concordance between MZ and DZ twins is similar, this indicates that variation in the phenotype is likely to be under shared environmental influence.

Using this information, univariate twin models decompose the variance in a measured phenotype into 3 different sources of variance: additive genetic influences (A), also known as heritability; shared environmental influences (C), which contribute to similarity within-twin pairs; and nonshared environmental influences (E), which contribute to differences between co-twins and include random measurement error. The classical twin design can be extended to establish the extent to which the same or different genetic and environmental influences are at play across different time points (e.g., FS intake in toddlerhood and middle childhood). This is useful for identifying how continuing and novel genetic and environmental influences contribute to stability and change in intakes of FS and NNS over time. Before deriving these etiological estimates, 3 sets of correlations are obtained from phenotypic models, which provide an indication of the relative influences. First, the cross-twin within-trait correlation provides an indication of the relative genetic and environmental influence on the *variance* (individual differences) in each trait at each age. For instance, if an MZ correlation is roughly double the DZ correlation, the trait includes genetic influence but no shared environmental influence. If, however, the MZ–DZ correlations are closer to a 1:1 ratio, the trait is under important shared environmental influence with no genetic effects. The extent to which the MZ correlation is <1.0 indicates the amount of nonshared environmental influence. Second, the cross-twin cross-time correlation provides an indication of genetic and environmental contributions to the *covariance* in these traits over time. The same principle as above is applied; if MZ–DZ correlations resemble a 2:1 ratio, the longitudinal relationship is likely to be explained by genetic influences. Lastly, the within-twin, cross-time correlation derived is the longitudinal phenotypic correlation over time.

The Cholesky decomposition model is particularly useful for estimating the genetic and environmental influences using longitudinal data and provides 95% confidence intervals (CIs) and goodness-of-fit data. It estimates univariate parameters at each age for additive genetic influences (A), shared environmental influences (C), and nonshared environmental influences (E), which also includes random measurement error. Covariance paths in this model indicate how much of the etiological influence (A, C, and E) is carried over from one time point to another (e.g., the same genes or environmental factors at play at both ages), which contributes to stability in the measured phenotype over time. The unique variance explained at each time point indicates how much of the etiological influence is not shared across time points, which contributes to the change in the measured phenotype over time. In this study, we therefore used the ACE Cholesky twin model (combining Additive genetic influences (A), Common/shared environmental influences (C) and Unique/non-shared environmental influences (E)) to examine: *1*) genetic and environmental contributions to variation in intake of FS and NNS in toddlerhood (21 mo) and middle childhood (7 y); and *2*) continuing and novel genetic and environmental influences contributing to stability and change in intakes of FS and NNS from toddlerhood to middle childhood.

FS intake was modeled as a continuous variable, with sex and corresponding age regressed, and residuals modeled. A threshold liability model was fitted for NNS (an ordinal variable), with age and sex as covariates on the thresholds. Threshold models assume that there is an underlying continuum of liability that is normally distributed in the population, and that variable categories (nonconsumer, low consumer, high consumer) are a result of 1 or more artificial divisions (thresholds) on this normal distribution. Analyses are therefore conducted on the underlying liability to the trait. To ease model specification, we began the threshold liability analysis with a constrained phenotypic model, which estimates one overall phenotypic correlation regardless of twin order and zygosity and symmetric cross-twin cross-trait correlations for MZ and DZ twins.

A fully saturated model, allowing all parameters to be estimated freely, was fitted for FS intake. We then fit submodels equating means and SDs across twin order and zygosity groups. For NNS intake, we fit submodels equating thresholds across twin order and zygosity. Following these constraints, we derived 3 types of correlations for both variables: *1*) within-twin cross-time correlations for FS and NNS intake at each time point for MZs and DZs separately (i.e., longitudinal phenotypic correlations); *2*) cross-twin cross-time correlations for MZ and DZ pairs separately (the basis of the longitudinal analyses); and *3*) cross-twin within-time correlations for MZ and DZ twins separately (the basis of the univariate analyses). Correlations and variable distributions were examined to determine suitability for biometric twin modeling (e.g., yielding correlations >0.2).

Following the full ACE model, we further tested submodels in which A and C paths were dropped separately (AE and CE models) and dropped together (E model). The E component is never dropped from the model because it includes random measurement error.

To detect best-fitting models, differences in minus twice log likelihood (–2LL) (distributed as χ^2^) were examined between nested models, in addition to Akaike Information Criterion (AIC), whereby a lower AIC generally indicates a better fit [37,38]. Twin model fitting was conducted using the OpenMx statistical package in R [39].

## Results

Descriptive statistics for the baseline and analysis sample are displayed in Table 1. Compared with the baseline sample, both dietary data samples had a significantly higher proportion of participants of White ethnic background, and both samples had a higher composite SES score. The samples were similar in all other characteristics. At 21 mo, toddlers consumed 1033 kcal/d (SD: 187); at 7 y, this was 1500 kcal/d (SD: 259). On average, participants at 21 mo consumed 9.3%EI from FS (SD: 4.6); at 7 y, this was 14.1%EI (SD: 4.9). There was a wide range of intake of FS at both time points, with FS intake contributing to 0.5%EI to 30.7%EI and 1.4%EI to 38.4%EI at 21 mo and 7 y, respectively. For NNS intake, at 21 mo, over half of the sample were classified as “nonconsumers” (*n* = 1634; 63.0%), 479 (18.5%) were classified as low consumers, and 479 (18.5%) were high consumers. At 7 y, 319 (53.9%) participants were classified as nonconsumers, 136 (23.0%) were low consumers, and 137 (23.1%) were high consumers. Intakes in both FS (*r* = 0.28, *P* < 0.001) and NNS (*r* = 0.35, *P* < 0.001) were significantly stable from 21 mo to 7 y, although correlations were small to moderate, indicating that there was greater change than stability in intakes over time.

**TABLE 1:** Dietary data sample characteristics at 21 mo and 7 y of age compared with Gemini baseline sample characteristics.

Characteristic	Baseline Gemini sample (*n* = 4804 twins; *n* = 2402 families)	21-mo dietary data sample (*n* = 2592 twins; *n* = 1296 families)	*P* value	7-y dietary data sample (*n* = 592 twins; *n* = 296 families)	*P* value
	*n* (%) or mean (SD)	*n* (%) or mean (SD)		*n* (%) or mean (SD)	
Sex					
Boys	2386 (49.7)	1277 (49.3)		292 (49.3)	
Girls	2418 (50.3)	1315 (50.7)	0.548^1^	300 (50.7)	0.859^1^
Ethnicity					
White	4462 (92.9)	2458 (94.8)		572 (96.6)	
Non-White	338 (7.0)	134 (5.2)	<0.001^1^	20 (3.4)	<0.001^1^
Not known	4 (0.1)	—		—	
SES^2^	4.3 (1.4)	4.6 (1.3)	<0.001^3^	4.9 (1.1)	<0.001^3^
Age at diary (mo)	—	20.9 (1.1)		85.3 (2.6)	
Gestational age (wk)	36.2 (2.5)	36.2 (2.5)	0.860	36.4 (2.4)	0.617
Weight at birth (kg)	2.5 (0.5)	2.5 (0.5)	0.466	2.5 (0.5)	0.368
Weight SD Score (SDS) at birth (kg)	–0.6 (0.9)	–0.6 (0.9)	0.536	–0.6 (0.9)	0.724

Abbreviation: SES, socioeconomic status.

1 Pearson’s χ^2^ test to compare categorical characteristics between the baseline Gemini sample and the dietary data sample. Significant differences (*P* < 0.05) are shown in bold.

2 Composite measure of SES [34]. Higher values represent increased SES outcomes.

3 Independent samples *t*-test differences in mean between baseline Gemini sample and dietary data sample. Significant differences (*P* < 0.05) are shown in bold.

The model-fit analyses for FS intake are shown in Supplementary Table 1; separate thresholds were modeled for NNS for MZ and DZ twins in the ACE model because equating thresholds across twin order and zygosity resulted in a significant reduction in fit. Intraclass correlations were very similar for DZ and MZ twin pairs for FS and NNS intake at 21 mo and for NNS intake at 7 y, indicating that a high proportion of variance in these measurements would be explained by the shared environment (Table 2). However, the slightly lower correlation between DZ pairs compared with MZ pairs for intake in FS intake at 7 y indicated some genetic contribution to variation as well.

**TABLE 2:** Phenotypic correlations for free sugar and NNS intake.

Variables	Cross-twin within trait	
	MZ	DZ
FS intake (%EI) at 21 mo (*N* = 2592)	**0.97 (0.97, 0.98)**	**0.93 (0.92, 0.94)**
FS intake (%EI) at 7 y (*N* = 592)	**0.92 (0.88, 0.94)**	**0.75 (0.68, 0.80)**
NNS intake at 21 mo (*N* = 2592)	**0.99 (0.97, 0.99)**	**0.99 (0.98, 0.99)**
NNS intake at 7 y (*N* = 592)	0.92 (–0.13, 0.92)	**0.96 (0.86, 0.99)**

Variables	Cross-twin cross time		Phenotypic correlation
FS intake (%EI) 21 mo—7 y	**0.28 (0.17, 0.37)**	**0.26 (0.16, 0.36)**	**0.28 (0.17, 0.37)**
NNS intake 21 mo—7 y	**0.33 (0.33, 0.65)**	**0.36 (0.34, 0.37)**	**0.35 (0.20, 0.36)**

Significant influences shown in bold, as indicated by 95% confidence interval not crossing zero.

Correlations between the NNS variables were polychoric, given the ordinal nature of the variable.

Abbreviations: %EI, as a percentage of total energy intake; DZ, dizygotic; FS, free sugars; MZ, monozygotic; NNS, nonnutritive sweeteners.

**FIGURE 1:**
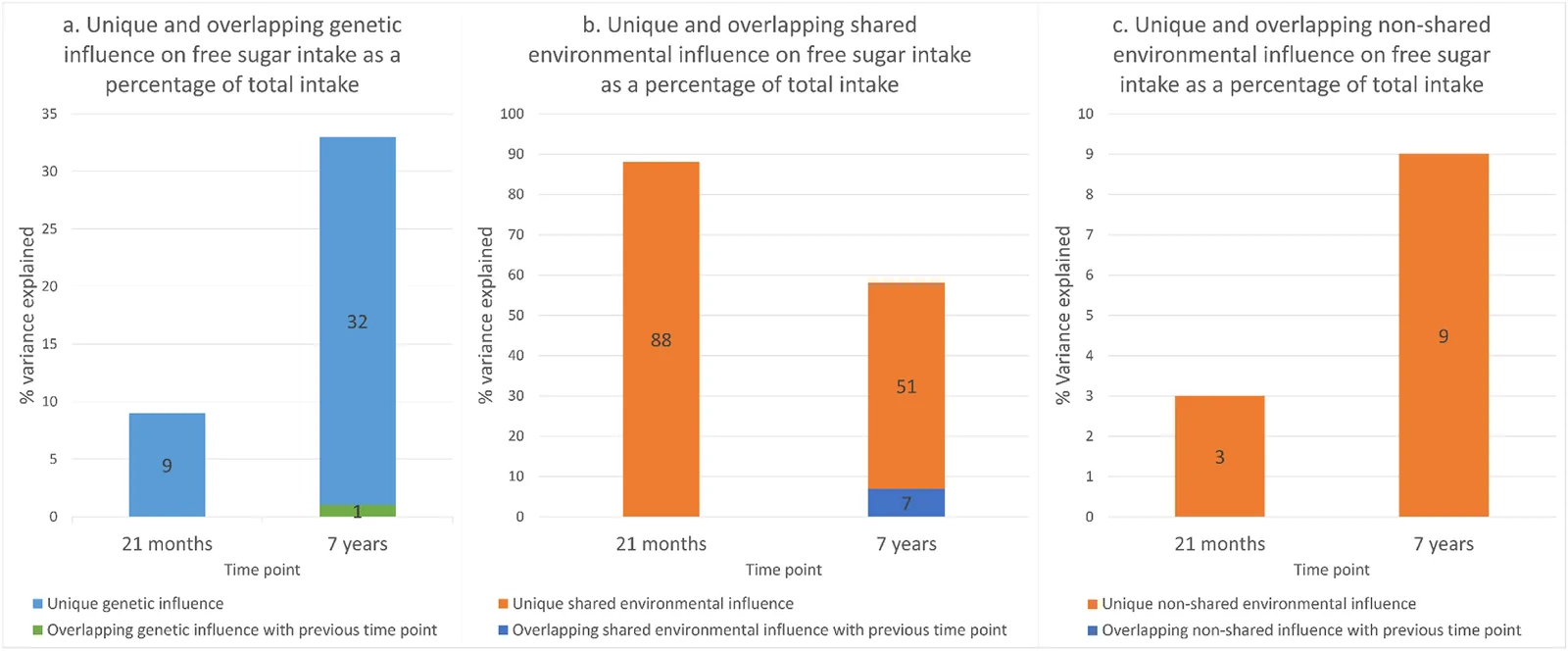
Unique and overlapping genetic and shared environmental effects on free sugar intake at 21 mo and 7 y. The y-axes for these graphs represent the total proportion of variance explained by genetic and environmental effects at that given time, and therefore do not add ≤100%. The total variance explained can be extracted by summing the proportion of variance explained at each age (e.g., for 21 mo, this will be 9% + 88% + 3% = 100%).

Results from the ACE model confirmed the etiology indicated by the patterns observed in the twin correlations (Table 3). Variation in FS intake was largely explained by shared environmental influences at 21 mo (88%; 95% CI: 86%, 90%) with a significantly lower, but also sizable, influence of the shared environment at 7 y (58%; 95% CI: 45%, 69%). Most of the shared environmental influences on FS intake at 7 y were novel—i.e., the majority of the shared environmental influences on FS intake at 7 y of age were different to those in operation at 21 mo of age (51% of the total 58%), whereas only 7% of the total (58%) shared environmental influences on intake of FS at 7 y were the same as those shaping intake at 21 mo (12% of the total shared environmental influence at 7 y of age—7% of 58%) (Figure 1). There was a small but significant genetic influence on variation in FS intake at 21 mo (9%; 95% CI: 8%, 11%), which increased significantly to a moderate genetic influence of 33% (95% CI: 22%, 47%) by 7 y of age. Virtually all the genetic influence at 7 y was unshared (32% of 33%) with that in operation at 21 mo of age—i.e., only 1% of the total (33%) genetic influence at 7 y was a continuation from 21 mo. A small but significant increase was also observed in unique environmental effects from 21 mo (3%; 95% CI: 2%, 3%) to 7 y (9%; 95% CI: 6%, 12%), but none of the nonshared environmental influences at 21 mo were still in operation at 7 y. Cholesky paths illustrating shared transmission paths between time points can be viewed in Supplementary Table 2.

**TABLE 3:** Total variance components for free sugar and NNS intake at 21 mo and 7 y.

Variable	Time point	A	C	E
FS intake (%EI)	21 mo	0.09 (0.08, 0.11)∗	0.88 (0.86, 0.90)∗	0.03 (0.02, 0.03)∗
	7 y	0.33 (0.22, 0.47)∗	0.58 (0.45, 0.69)∗	0.09 (0.06, 0.12)∗
NNS	21 mo	0.00 (0.00, 0.01)	0.99 (0.99, 1)∗	0.01 (0.00, 0.01)
	7 y	0.03 (0.00, 0.13)	0.92 (0.86, 1)∗	0.05 (0.03, 0.10)∗

∗Significant influences as indicated by 95% confidence interval not crossing zero.

Abbreviations: %EI, as a percentage of total energy intake; A, additive genetic influences; C, common/shared environmental influences; E, unique environmental influences; FS, free sugars; NNS, nonnutritive sweeteners.

**FIGURE 2:**
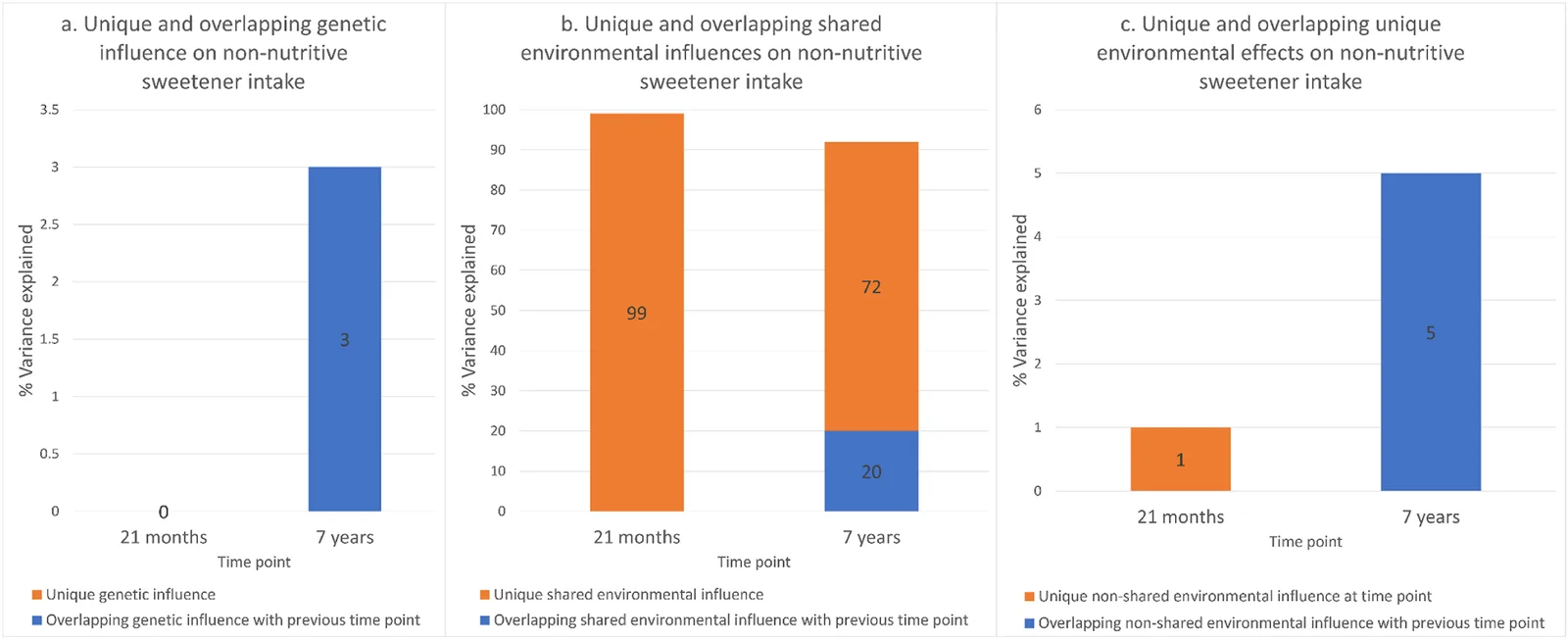
Unique and overlapping genetic and shared environmental effects on nonnutritive sweetener intake at 21 mo and 7 y. The y-axes for these graphs represent the total proportion of variance explained by genetic and environmental effects at that given time, and therefore do not add ≤100%. The total variance explained can be extracted by summing the proportion of variance explained at each age (e.g., for 7 y, this will be 3% + 92% + 5% = 100%).

Variation in NNS intake was explained, almost entirely, by shared environmental influences at both 21 mo (99%; 95% CI: 99%, 100%) and 7 y (92%; 95% CI: 86%, 100%), and there was no significant difference in the size of the effects at either age. As with FS intake, most shared environmental influence at 7 y (72%/92%) was novel (i.e., not the same as the shared environmental influences at 21 mo), although 20% of the variance (i.e., 21.7% of the total shared environmental influence) was overlapping with 21 mo (Figure 2). The remaining variance in NNS intake at 21 mo was explained by the unique environment (1%; 95% CI: 0%, 1%), which had increased slightly but significantly by 7 y (5%; 95% CI: 3%, 10%). None of the nonshared environmental influences across the 2 ages were the same (Supplementary Table 2). There was no significant genetic influence on variation in NNS intake at either 21 mo (0%; 95% CI: 0%, 1%) or 7 y (3%; 95% CI: 0%, 13%).

## Discussion

This is the first study to estimate the relative contribution of genetic and environmental factors to individual differences in FS and NNS intakes during toddlerhood and middle childhood, and to investigate stability and change in intakes over time. Shared environmental factors were the dominant drivers of variation in intakes of FS and NNS in toddlerhood, with this strong environmental influence persisting for NNS intake into middle childhood. In contrast, genetic factors became more prominent in shaping variation in FS intake as children aged. Shared environmental influences play a significant role in shaping FS and NNS intakes in both toddlerhood and middle childhood. Toddlers and young children have limited independence. The environmental factors twins encounter together in the home, such as parental feeding practices and regulations around sugary food and drink consumption, or drinks containing NNS, are key influencers of intake [[40], [41], [42]]. Caregivers construct the home food environment, determining food availability, portion sizes, and feeding practices, such as offering sweet foods as rewards or snacks [43,44]. Macrolevel environmental factors that could influence FS and NNS intake, shared by twin pairs, include familial SES, neighborhood environment, schoolwide policies, and cultural norms [[45], [46], [47]]. Added sugars and sugar-sweetened beverages (SSBs) both contribute to total FS intake and have been inversely associated with SES markers, including family income, education, and deprivation [[48], [49], [50]]. Consequently, any genetic variation that could potentially influence differential intake of these sweet dietary constituents in a more permissive setting may be masked by caregiver choices [23].

There was some stability in intakes of FS and NNS over time, driven almost entirely by continuing shared environmental influences between these 2 developmental periods. This suggests enduring and consistent parental influence over the home food environment, with children likely exposed to similar foods and beverages in the home from toddlerhood to middle childhood [[45], [46], [47], [48], [49], [50]], or the continuation of macrolevel factors such as the quality of the neighborhood food environment. However, more change was observed over time than stability, and much of this was also driven by novel shared environmental factors emerging over time. At 7 y, most of the shared environmental influences contributing to the variation in NNS intake were novel (72%), indicating the emergence of new influential environmental factors, emphasizing the dynamic nature of children’s shared environments. As children age, they spend more time outside the family home, and caregiver control over diet gradually diminishes [51]. Twins typically attend the same school, resulting in similar exposure to common school food environments, likely partly replacing some of the influence of the shared home or nursery environment present during toddlerhood.

Findings indicated that genetic factors influencing intake of foods and beverages high in FS become more pronounced as children mature, although genetic factors did not influence NNS intake. During toddlerhood, a significant but very low genetic influence (9%) on variation in FS intake was observed. However, primary caregivers exert substantial control over toddlers’ diets, limiting opportunity for genetic expression. By 7 y, FS intake heritability had increased significantly and substantially to 33%. Although there are no other longitudinal twin studies on FS intake across childhood, these findings align with 2 cross-sectional twin studies exploring variation in intake of added sugar and sugary food consumption during similar developmental windows [23,25]. The higher heritability observed at 7 y indicates growing autonomy in dietary choices as children mature. Increased exposure to permissive food environments, coupled with less parental control, likely enables greater expression of genetically determined factors influencing variation in FS intake, such as preferences for sweetness [[52], [53], [54]].

Our findings regarding NNS highlight the dominant role of environmental influences in shaping intake in early childhood with minimal contribution from genetic factors. In this study, total dietary intake of FS is examined, whereas NNS intake is derived from non-nutritively sweetened beverages (NNSB) only. In early childhood, children are likely too young to choose NNSB for health reasons; it is plausible intake is more likely shaped by parental and later school-level decisions around beverage availability [55,56]. This is supported by United Kingdom household data showing children were almost 5 times more likely to consume NNSB if their parents were consumers [57]. These findings hold significant implications for the development of public health policies around intake of sugar and NNS in toddlerhood and middle childhood. The results emphasize that public health policies and interventions targeting FS and NNS intake during toddlerhood should focus on aspects of the shared environment, such as the family home or nursery food environment and parental or caregiver behaviors. The shift in the relative influence of genetic and environmental factors as toddlers mature into middle childhood emphasizes the need to consider developmental transitions when targeting dietary intake in children. This suggests that public health interventions and policies aiming to reduce sugar intake in childhood may be most effective when they are age-specific and consider the different influences on intakes of FS for toddlers compared with older children.

In middle childhood, although the home environment remains important, refinement of whole school approaches aiming to reduce disparities and improve food quality, availability, and knowledge around sugar consumption may be useful [58]. Targeted food industry legislation could also benefit population-wide attempts to reduce sugar consumption. The United Kingdom’s Soft Drinks Industry Levy, implemented in April 2018, has already shown promise as a successful population-level nutritional intervention for the reduction of sugar intake from SSBs [59]. Expansion of this policy to encourage shifts within industry to produce lower sugar products may be beneficial for further reductions in sugar purchasing—and ultimately intake—across the United Kingdom. Given that the Gemini sample comprises young children, consumption patterns of SSBs may differ from those seen in adolescent/young adult populations. Future work examining the relative genetic and environmental influences across the most consumed dietary sources of FS by young children may help identify those more strongly shaped by shared environmental factors, which could support more targeted reformulation efforts and public health policies to improve childhood nutrition.

Higher genetic influence on FS intake in older children may make it harder to intervene on FS intake at 7 y than at 21-mo. By 7 y of age, intake is already under moderate genetic influence, with some children likely expressing stronger desires for these foods than others, making intervention increasingly challenging. Nevertheless, the findings related to variation in NNSB intake show that it is possible for strong environmental influences over intakes of certain beverages to persist into middle childhood. If similarly, stringent environmental policies around FS intake could be achieved, it is possible that genetic expression in FS intake could be suppressed into middle childhood and beyond.

A notable strength of this study was that Gemini includes one of the largest contemporary population-based dietary datasets of United Kingdom toddlers, offering prospective insight across key developmental windows. However, a comparatively smaller sample was available at 7 y of age (23% of the 21-mo sample), which may limit generalizability. Although dietary intake was collected using detailed 3-d diet diaries, parent-reported dietary intake data may still be subject to reporting bias [60]. FS and NNS intake are investigated using specialist intake estimation methods, one of which was developed by the current research team [30,33]. However, finding comparability is limited in that FS intake was based on complete dietary intake, whereas NNS intake was estimated from beverages only. Although <1% of all individual items consumed across both toddlerhood and middle childhood were identified as foods containing NNS (e.g., low-energy ice cream), this contribution was not considered [33]. As only one-third of toddlers were NNS consumers, the data were modeled using a threshold model, which may have reduced sensitivity to smaller within-pair differences and inflated shared environmental estimates. The Gemini cohort at baseline was representative of families in England and Wales for sex, gestational age, zygosity, and birth weight, although they were of higher SES and had a higher percentage of White mothers (87%). This difference is particularly apparent for the 7-y data, which included only a subsample of highly engaged families invited to contribute data. Findings may therefore not be generalizable to broader United Kingdom populations. Lastly, twin analyses assume that both MZ and DZ twins are exposed to the shared environment to a similar extent. Several studies have tested the equal environment assumption and, overall, the literature suggests that no violation of this assumption for most complex traits [[61], [62], [63]]. In addition, nonshared environmental estimates capture random measurement error, but these were particularly low in the current study. This indicates that measurement error was likely to be shared within-twin pairs, which could have inflated shared environmental estimates. Although twin studies estimate the total influence from genetic and environmental factors, they do not provide specific insight into the actual genetic and environmental factors involved. Therefore, epidemiological studies that examine associations between specific environmental factors and genomic studies that examine associations between measured genetic variants are needed to identify the actual environmental and genetic factors contributing to the observed effects.

In conclusion, shared environmental influences are the most important determinant of individual differences in both FS and NNS intake during early life. There is an important developmental change in influences on FS intake over time, characterized by a sizable increase in genetic influence and a diminishing influence of the shared environment as toddlers mature into older children. In contrast, individual differences in NNS intake were predominantly shaped by shared environmental factors across both ages, with minimal evidence of genetic contribution.

## Author contributions

The authors’ responsibilities were as follows – LH, ZN, CHL: designed and conducted the study; ZN: performed statistical analysis and interpreted the findings; LH: wrote the first draft of the manuscript; RC, AF, AS, EF, JCGH, JAH, AR, CHL: interpreted the results, critically revised the manuscript, and provided intellectual content; CHL: obtained funding; and all authors: read and approved the final manuscript.

## Data availability

Data described in the manuscript, code book, and analytic code may be made available upon request, pending approval by the Gemini study team.

## Funding

Cancer Research UK funded the first 5 years of data collection (from 2007-2012), including the 21 months dietary data collection (grant number: 161722). A Medical Research Council (MRC) PhD studentship funded the 7 years dietary data collection (grant number: 160897), and a Horizon 2020 grant funded coding of the dietary data for free sugar and NNS, and LH's PhD studentship (EU H2020 grant number: 774293).

## Declaration of generative AI and AI-assisted technologies in the writing process

The author(s) declare that no generative AI or AI-assisted technologies were used in the writing of this manuscript.

## Conflict of interest

AR has received honoraria from Nestlé, Unilever, and the International Sweeteners Association. JCGH and JAH have received research funding from the American Beverage Association in respect to a study examining the impact. The other authors report no conflicts of interest.
