# Supplementary material for: Genetic and Environmental Influences on Free Sugar and Nonnutritive Sweetener Intake in Toddlerhood and Middle Childhood Using Data from the Gemini Twin Cohort: Findings from the SWEET Project
Source: J Nutr. 2026 May 8;156(7):101586. doi: 10.1016/j.tjnut.2026.101586 (PMC13494194; doi:10.1016/j.tjnut.2026.101586)
Supplement: Supplementary file 1 [file mmc1.docx]

**Genetic and environmental influences on free sugar and non-nutritive sweetener intake in toddlerhood and middle-childhood using data from the Gemini twin cohort: findings from the SWEET project**

**Lisa Heggie, Zeynep Nas et al**

Supplementary file, Table 1. Fit statistics of longitudinal models

| **Variable** | **Model** | **Ep** | **-2LL** | **df** | **AIC** | **ΔLL** | **Δdf** | **p** |
| --- | --- | --- | --- | --- | --- | --- | --- | --- |
| Free sugar intake 21 months – 7 years (longitudinal model) | Saturated | 28 | 15264.55 | 3108 | 15320.55 | - | - | - |
|  | Sub1a | 20 | 15272.77 | 3116 | 15312.77 | 8.22 | 8 | 0.41 |
|  | Sub1b | 11 | 15286.89 | 3125 | 15308.89 | 22.33 | 17 | 0.17 |
|  | ACE | 11 | 15286.89 | 3125 | 15308.89 | 22.33 | 17 | 0.17 |
|  | AE | 8 | 16277.26 | 3128 | 16293.26 | 990.37 | 3 | 2.21e-214 |
|  | CE | 8 | 15447.66 | 3128 | 15463.66 | 160.78 | 3 | 1.25e-34 |
|  | E | 5 | 18543.16 | 3131 | 18553.16 | 3256.27 | 6 | <.001 |
| NNS 21 months – 7 years (longitudinal model) | Saturated | 19 | 3627.97 | 3117 | 3665.97 | - | - | - |
|  | SubTM1 | 17 | 3774.06 | 3119 | 3808.06 | 146.10 | 2 | <.01 |
|  | SubTM2 | 17 | 3635.81 | 3119 | 3669.81 | 7.84 | 2 | .02 |
|  | SubTM3 | 15 | 3668.11 | 3121 | 3698.11 | 40.14 | 4 | <.01 |
|  | ACE | 21 | 3598.95 | 3119 | 3640.95 | -29.02 | 2 | 1 |
|  | AE | 18 | 4447.47 | 3122 | 4483.47 | 848.51 | 3 | 1.30e-187 |
|  | CE | 14 | 3601.20 | 3122 | 3637.20 | 2.24 | 3 | 0.52 |
|  | E | 11 | 5780.87 | 3125 | 5810.87 | 2181.91 | 6 | <.001 |

*Note.* A liability threshold model was run for non-nutritive sweetener intake.

Sub 1a = Equating means and variances across twin order; Sub 1b = Equating means and variances across twin order and zygosity. SubTM 1= equating Thresholds across Twin 1 and Twin 2 within MZ group. SubTM 2 = equating Thresholds across Twin 1 and Twin 2 within DZ group. SubTM 3= equating Thresholds across Twin 1 and Twin 2 and zygosity groups. Sub models compared to fully saturated model. ACE models compared to fully saturated. AE = Model dropping C influences; CE= Model dropping A influences; E= Model dropping both A and C influences.

**Supplementary file, Table 2. Cholesky paths between time points for free sugar (FS) and non-nutritive sweetener (NNS) intake**

| Variable | A Cholesky | C Cholesky | E Cholesky |
| --- | --- | --- | --- |
| FS intake (%EI) | .01 (.00, .06) | **.07 (.02, .14)** | .00 (.00, .01) |
| NNS | .03 (.00, .03) | **.20 (.11, .22)** | **.05 (.04, .06)** |

*Note.* Cholesky paths indicate the degree of shared genetic and environmental transmission between time points. Significant influences shown in bold, as indicated by 95%CI not crossing zero. %EI, as a percentage of total energy intake. FS, free sugars. NNS, non-nutritive sweeteners.

Supplementary file, Figure 1. Gemini participant flow diagram and inclusion in age-specific (21 months and 7 years) and longitudinal analyses

**Families enrolled in the Gemini cohort at baseline**
n = 2,402 families (4,804 twins)

**Participants with genetic data and ≥2 days of dietary data at 21 months**
n = 1,357 families (2,592 twins)

**Participants with genetic data and ≥2 days of dietary data at 7 years**
n = 309 families (592 twins)

*Included in age-specific analyses at 21 months*

*Included in age-specific analyses at 7 years and longitudinal analyses*
